# Prescribing trends of gabapentin, pregabalin, and oxycodone: a secondary analysis of primary care prescribing patterns in England

**DOI:** 10.3399/bjgpopen19X101662

**Published:** 2019-09-18

**Authors:** Katlyn Green, Nora Cooke O'Dowd, Hilary Watt, Azeem Majeed, Richard J Pinder

**Affiliations:** 1 Analyst, Public Private Ltd, London, UK; 2 Public Health Intelligence Analyst, Public Health Division, Southwark Council, London, UK; 3 Consultant Statistician, Department of Primary Care and Public Health, Imperial College London, London, UK; 4 Professor of Primary Care, Department of Primary Care and Public Health, Imperial College London, London, UK; 5 Consultant in Public Health Medicine, Department of Primary Care and Public Health, Imperial College London, London, UK

**Keywords:** oxycodone, gabapentin, pregabalin, prescribing, misuse

## Abstract

**Background:**

The risk of iatrogenic harm from the use and misuse of prescription drugs such as gabapentin, pregabalin, and oxycodone is substantial. In recent years, deaths associated with these drugs in England have increased.

**Aim:**

To characterise general practice prescribing trends for gabapentin, pregabalin, and oxycodone — termed dependence forming medicines (DFM) — in England and describe potential drivers of unwarranted variation (that is, very high prescribing).

**Design & setting:**

This study is a retrospective secondary analysis of open source, publicly available government data from various sources pertaining to primary care demographics and prescriptions.

**Method:**

This study used 5 consecutive years (April 2013–March 2018) of aggregate data to investigate longitudinal trends of prescribing and variation in prescribing trends at practice and clinical commissioning group (CCG) level.

**Results:**

Annual prescriptions of gabapentin, pregabalin, and oxycodone increased each year over the period. Variation in prescribing trends was associated with GP practice deprivation quintile, where the most deprived GP practices prescribed 313% (*P*<0.001) and 238% (*P*<0.001) greater volumes of gabapentin and pregabalin per person respectively, than practices in the least deprived quintile. The highest prescribing CCGs of each of these drugs were predominantly in northern and eastern regions of England.

**Conclusion:**

Substantial increases in gabapentin, pregabalin, and oxycodone prescriptions are concerning and will increase iatrogenic harm from drug-related morbidity and mortality. More research is needed to understand the large variation in prescribing between general practices, and to develop and implement interventions to reduce unwarranted variation and increase the appropriateness of prescribing of these drugs.

## How this fits in

Prescription drugs can cause iatrogenic harm in the form of misuse, dependence, and poisonings. In the US, the opioid crisis is a serious public health problem and was driven in part by increasing opioid prescriptions. In the UK, rates of opioid and GABA-ergic medication (GABA analogues) use are known to be increasing, with a corresponding increase in reports of related harms, such as mortality and morbidity. It is necessary to investigate these trends further, and whether deprivation is driving increased rates at a GP practice population and CCG level.

## Introduction

Analgesics are prescribed widely in primary care to reduce the burden of pain and improve quality of life. Opioids are among the most potent painkillers, but have well-established risks of misuse and addiction. Other drugs, gabapentin and pregabalin, are also widely used as painkillers, albeit with historically fewer concerns about their risks. The ongoing US opioid crisis — which was preceded by decades of rising prescribing rates of drugs such as oxycontin (oxycodone),^1^ among a myriad of wider drivers^2^ — claims an estimated 115 lives a day, at an approximate annual cost to the US economy of $78.5 billion,^3^ and disproportionately affects deprived areas of the country.^4^ In the UK and particularly England, concern has emerged in recent years about an increase in opioid prescribing for chronic pain, which constitutes the largest bulk of prescribing. This is despite opioids’ lack of efficacy in managing chronic pain over the long-term,^5^ and despite associated risks.^6^ There is uncertainty about the efficacy of GABA-ergic medications for long-term chronic pain management and most new prescriptions for this use are made off-label.^7^

Amid existing concerns about the prescription and management of opioids in primary care, there is also now growing concern about the risks posed by gabapentin and pregabalin medications. This is demonstrated by a wide-ranging report into the Diversion and Illicit Supply of Medicines by the Advisory Council on the Misuse of Drugs in the UK.^6^ The findings indicated a growing problem with gabapentin and pregabalin medication diversion and misuse, particularly in people with a history of substance misuse. In 2017, the Home Office undertook a consultation on the scheduling of gabapentin and pregabalin medications to classify them as Class C medications, enacted in April 2019.^8^ Oxycodone is an opioid with known risk profile and is a Class A medication, subject to substantial legislative controls. In light of growing evidence and perception of increased prescribing of these drugs, Public Health England (PHE) has begun a review on the topic.^9^

There is a growing body of literature characterising prescribing behaviours and risks of harm to the national population. A recent comprehensive retrospective database study using open data sources found that, overall, primary care opioid prescriptions increased by 34% in England between 1998 and 2016.^10^ When total oral morphine equivalency, an experimental measure of relative oral morphine dose for all opioids to quantify risk of harm, is accounted for, the increase was 127%.^10^ This study found considerable variation for opioid prescribing associated with deprivation, by CCG and GP practice list size.

Another study in the UK reported a rise in prescribing of DFM from 2000 to 2015 using data from the Clinical Practice Research Database.^11^ Opioids, antidepressants, Z-drugs, and gabapentin and pregabalin medications have seen increases over this timeframe. In 2015, opioids were prescribed to 5% of the study population of non-cancer and non-palliative care patients. Overall, 9% of patients were prescribed some form of DFM in 2015, an increase from 5% in 2000.^11^ The duration of prescription for pregabalin, gabapentin, and oxycodone increased over the study period.

In 2013, a series of case reports highlighted the recreational use of pregabalin and gabapentin in patients with histories of substance misuse in Belfast that led to emergency department admission.^12^ A recent study in England asserted higher opioid dosages increased the risk of harm to individuals and found correlations between a higher active ingredient (per mg per morphine per head per CCG) and social deprivation scores (Index of Multiple Deprivation [IMD] scores) and geographical locations. This study identified substantial increases in prescriptions of five of the eight most commonly prescribed opioids, including oxycodone, and the highest rate of prescribing in the North of England in 2010–2014.^13^ In the UK, from 2011 to 2016, drug poisoning deaths that named opioids and gabapentin and pregabalin medications have increased each year, though absolute number of deaths remains low.^14^ In 2011, pregabalin was named on four drug-related death certificates, and in 2016 that number had increased to 111.^14^ Death certificates naming oxycodone have also increased over time, but remain lower than other opioids (*n* = 75 in 2016).^14^

Increases in prescribing of gabapentin and pregabalin is also harmful due to the potential for polypharmacy with opioids. A Canadian case–control study illustrated that patients receiving opioid prescriptions saw an increased risk of opioid-related death when they also received gabapentin.^15^ These findings reflect a similar trend in England, whereby increased risk to heroin users of opioid-related death by overdose correlated with increases of prescribing of pregabalin and gabapentin from 2004 to 2013. Qualitative findings from interviews with heroin users report pregabalin is commonly found and used as a street drug with this population.^16^

The aim of this study was to investigate primary care prescribing patterns of gabapentin, pregabalin, and oxycodone in England, with a view to determining whether there is a potential public health problem.

## Method

### Data sources and collation

Prescribing data were retrieved from the primary care datasets collated and aggregated at practice-level each month by NHS Digital for all general practices in England.^17^ These data relate to dispensed prescriptions. All prescriptions relating to gabapentin, pregabalin, and oxycodone were identified by *British National Formulary* codes (available from the authors on request) and these were extracted over the 5 financial years from April 2013 to March 2018. The dataset includes every dosage and pack size available of the drugs, and so the active drug volume varies by prescription.

Using NHS England practice codes and drawing on nationally published general practice data, practice characteristics (list size, patient age, and sex distribution) taken from April of each year were prospectively applied for the subsequent 11 months.^18^ IMD scores (2015) by practice were extracted from PHE’s National General Practice Profiles^19^ and were categorised into quintiles (where 1 = least deprived). Quintile thresholds were set using lower super output area (LSOA) national ranking deciles published by the Office for National Statistics.^20^

To mitigate against partial year practice closures and other statistical artefacts, inclusion criteria for practices were specified a priori. Only practices with a prescribing setting in a GP practice as defined by the NHS Organisation Data Service (setting code 4), a list size >800 persons, open for the entirety of the financial year and with one or more of the selected drugs dispensed were included in the analysis. A convenience sample of excluded practices was taken to assess suitability of exclusion against the criteria. In doing this, a novel and comprehensive dataset was developed based on linkage of these multiple publicly available datasets. A flow diagram to demonstrate the data linkage process to develop the dataset is available from the authors on request.

Clinical commissioning group (CCG) list size and prescription volumes were calculated by summing GP practice data by associated CCG code. During the study period, some CCGs merged or were subject to geographical boundary changes.^21^ These areas and the practices affected were excluded from CCG-related analyses (available from the authors on request).

### Analytical approach

The combined database was used to undertake descriptive and inferential analysis using hypotheses specified a priori using two-tailed tests. Descriptive data are presented as median with 95% confidence intervals (CI). Associations between annual number of prescriptions per practice and IMD quintile were tested using negative binomial regression models. The dependent variable was the number of prescriptions of gabapentin, pregabalin, or oxycodone. The multivariate model was adjusted for financial year as a categorical variable and GP practice demographics. These demographics were practice list population, age, and sex. Age was included as count of registered patients aged 16–64 years and ≥65 years as percentages of the total GP list size. Children aged <16 years were excluded from the dataset. For sex, counts of females as a percentage of the total GP list size were used. The output of unadjusted and adjusted model, the β coefficients, were exponentiated to give the rate ratio (RRs) and 95% CI. All data were collated and analysed using Stata (STATA/SE version 13.1) and Microsoft Excel (2016).

CCGs were mapped using mean prescriptions per capita for 2017/2018. CCGs were categorised into categories of >1.5, 0.5–1.5, and <0.5 times the mean rate using MapInfo (Pro Version 15.0).

## Results

The number of GP practices included in the analyses decreased each year (Table 1); by 2017/18, there were 7% fewer GP practices than in 2013/2014. Correspondingly, the median list size of GP practices increased (Table 2).

**Table 1.** Total number of GP practices in England and number of GP practices included in this study

	**2013**	**2014**	**2015**	**2016**	**2017**
**Number of GP practices^a^**	8106	8002	7816	7680	7492
**Number of GP practices included**	7132	7029	6907	6728	6632

^a^Total number extracted from NHS Digital GP practice demographics.

**Table 2.** Median GP practice list size, median rate of gabapentin, pregabalin, and oxycodone prescriptions per 1000 population per GP practice

	**2013 Median (95% CI)**	**2014 Median (95% CI)**	**2015 Median (95% CI**	**2016 Median (95% CI)**	**2017 Median (95% CI)**
**GP practice list size**	5022 (4899 to 5096)	5100 (4935 to 5141)	5283 (5096 to 5300)	5478 (5285 to 5485)	5760 (5538 to 5772)
**Gabapentin prescriptions per 1000 population**	83.6(82.4 to 85.3)	98.7 (97.2 to 100.4)	113.3 (110.9 to 115.4)	127.2 (124.7 to 129.7)	136.2 (133 .3 to 138.5)
**Pregabalin prescriptions per 1000 population**	65.8 (64.3 to 66.8)	79.8 (78.6 to 81.4)	92.9 (91.2 to 94.9)	106.9 (104.9 to 108.8)	118.6 (116.8 to 120.2)
**Oxycodone prescriptions per 1000 population**	2.1 (2.0 to 2.1)	2.3 (2.2 to 2.3)	2.5 (2.4 to 2.7)	2.7 (2.6 to 2.8)	2.8 (2.8 to 2.9)

Year-on-year, the median rate of prescriptions per 1000 population for gabapentin, pregabalin, and oxycodone increased (Table 2). The largest increase was seen in pregabalin; in 2017/2018 the median number of prescriptions (119 per 1000 population) increased 1.8 times since 2013/2014. Overall, gabapentin remains the most prescribed drug (of the three) per 1000 population.

**Figure 1.**
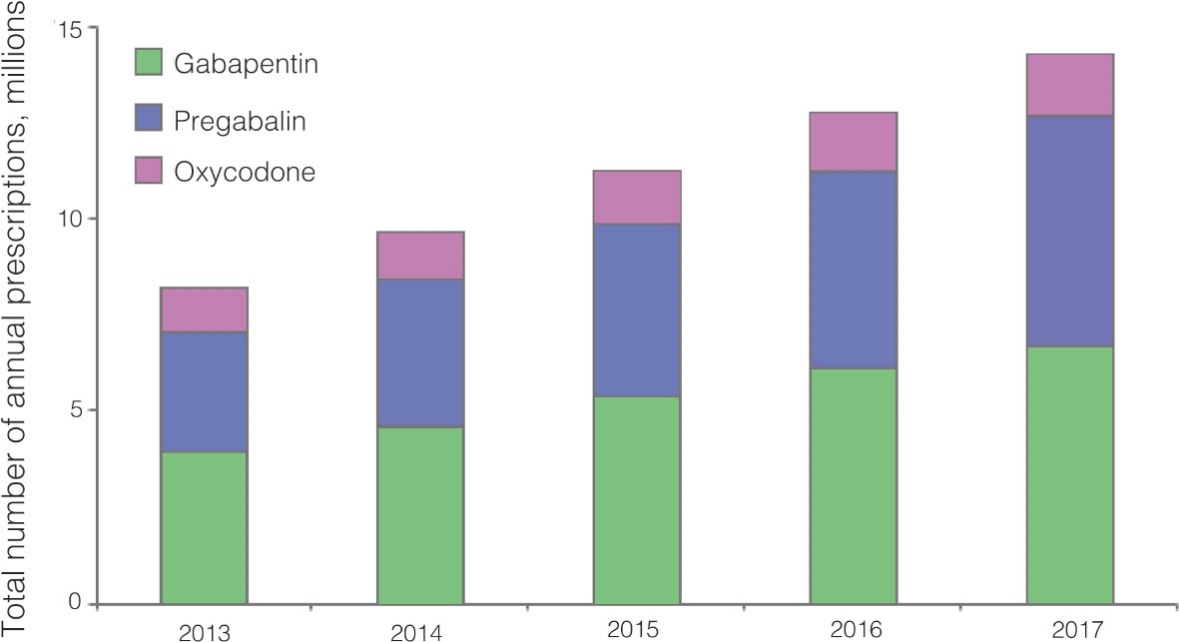
Absolute total number of prescriptions (in millions) of gabapentin, pregabalin, and oxycodone made in primary care per year.

The total number of prescriptions are increasing in line with the median number of prescriptions per 1000 population of each of the drugs (Figure 1). The absolute number of prescriptions made each year of oxycodone remains low (1.1 million in 2013/2014) in comparison to pregabalin (3.13 million), and gabapentin (3.93 million in 2013/2014), but overall there are substantial increases in the total number of prescriptions of gabapentin (6.72 million in 2017/2018), pregabalin (5.95 million in 2017/2018), and oxycodone (1.61 million in 2017/2018) over 5 years.

The inferential analysis (Table 3) shows a positive association between prescribing per person and deprivation, with more deprived areas recording substantially higher and significantly increased rates of prescribing for gabapentin, pregabalin, and oxycodone (*P*<0.001). This association remains significant (*P*<0.001) and accounts for more statistical variation in the multivariate analysis when adjusted for GP practice population age, and sex distribution and financial year.

**Table 3.** Association between number of prescriptions per person per GP practice and GP practice IMD quintile

	**Gabapentin**	**Pregabalin**	**Oxycodone**
**Univariate**	**RR 95% CI**	***P* value**	**RR (95% CI)**	***P* value**	**RR**	***P* value**
**IMD quintile**			
(least deprived) 1	1 (Ref)	Ref	1 (Ref)	Ref	1 (Ref)	Ref
2	1.26 (1.22 to 1.31)	<0.001	1.2 (1.16 to 1.24)	<0.001	1.19 (1.13 to 1.25)	<0.001
3	1.53 (1.48 to 1.58)	<0.001	1.35 (1.31 to 1.40)	<0.001	1.37 (1.31 to 1.44)	<0.001
4	1.8 (1.745 to 1.86)	<0.001	1.56 (1.51 to 1.61)	<0.001	1.47 (1.40 to 1.54)	<0.001
(most deprived) 5	2.29 (2.22 to 2.37)	<0.001	1.86 (1.80 to 1.92)	<0.001	1.71 (1.63 to 1.80)	<0.001
**Multivariate**						
**IMD quintile**						
(least deprived) 1	1 (Ref)	Ref	1 (Ref)	Ref	1 (Ref)	Ref
2	1.24 (1.21 to 1.28)	<0.001	1.2 (1.16 to 1.23)	<0.001	1.2 (1.15 to 1.25)	<0.001
3	1.59 (1.54 to 1.63)	<0.001	1.39 (1.35 to 1.44)	<0.001	1.47 (1.40 to 1.53)	<0.001
4	2.12 (2.06 to 2.18)	<0.001	1.79 (1.74 to 1.845)	<0.001	1.82 (1.74 to 1.90)	<0.001
(most deprived) 5	3.13 (3.04 to 3.23)	<0.001	2.38 (2.30 to 2.45)	<0.001	2.54 (2.42 to 2.66)	<0.001
**Year**						
2013	1 (Ref)	Ref	1 (Ref)	Ref	1 (Ref)	Ref
2014	1.2 (1.175 to 1.22)	<0.001	1.24 (1.21 to 1.26)	<0.001	1.11 (1.07 to 1.14)	<0.001
2015	1.36 (1.33 to 1.38)	<0.001	1.42 (1.40 to 1.45)	<0.001	1.19 (1.16 to 1.22)	<0.001
2016	1.51 (1.48 to 1.54)	<0.001	1.62 (1.59 to 1.655)	<0.001	1.26 (1.225 to 1.30)	<0.001
2017	1.55 (1.525 to 1.58)	<0.001	1.75 (1.72 to 1.79)	<0.001	1.27 (1.23 to 1.31)	<0.001
**Sex**						
% female	1 (0.10 to 1.00)	0.088	1 (0.10 to 1.00)	0.201	1.02 (1.02 to 1.03)	<0.001
**Age**						
% ≥65 years	1.04 (1.04 to 1.04)	<0.001	1.03 (1.03 to 1.03)	<0.001	1.04 (1.04 to 1.04)	<0.001

IMD = Index of Multiple Deprivation. Ref = reference. RR = rate ratio.

In England, GP practices located within the most deprived quintiles issue 313%, 238%, and 254% more prescriptions per head per GP practice of gabapentin, pregabalin, and oxycodone respectively than those in the least deprived quintile.

**Figure 2.**
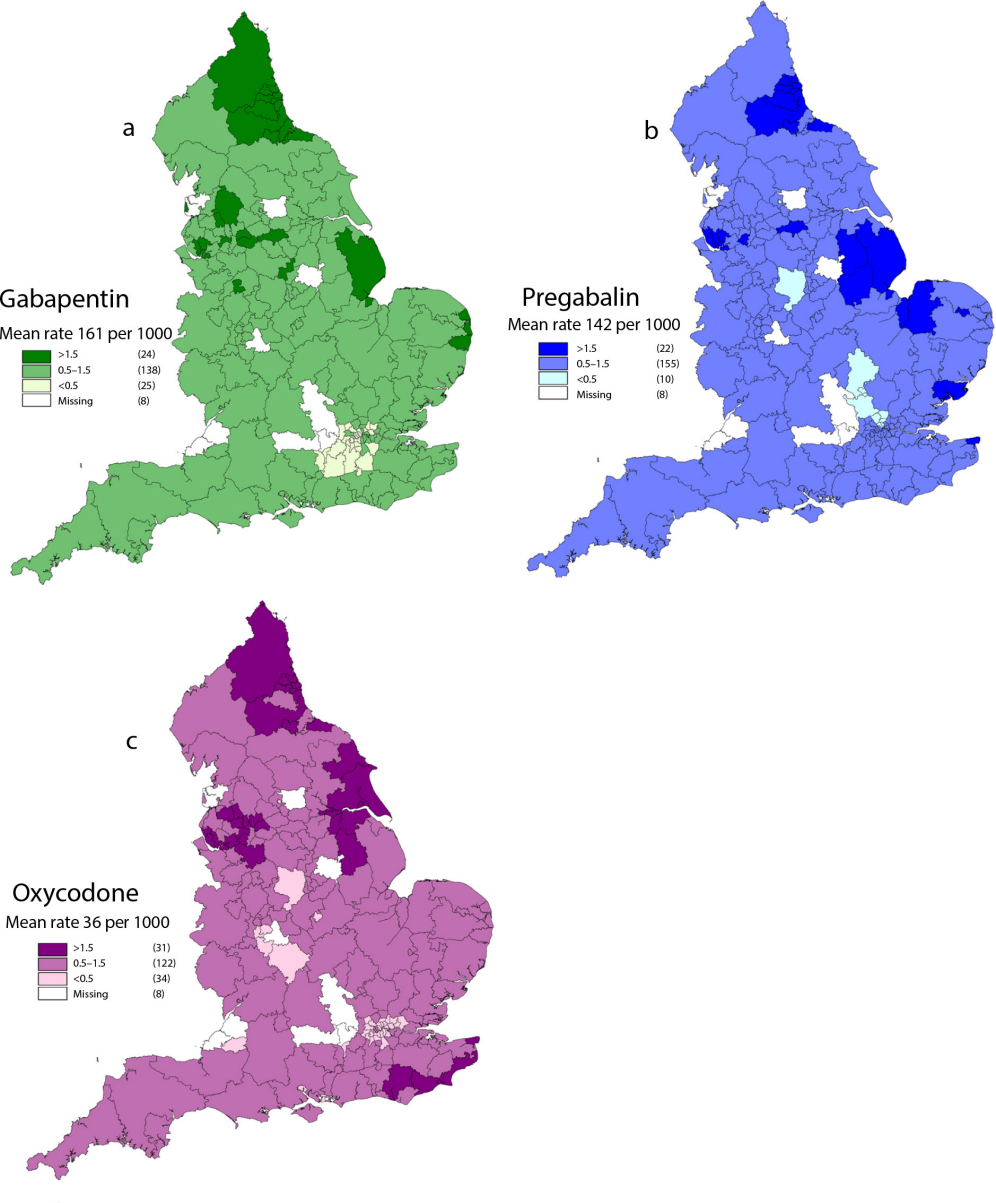
GIS visualisation of CCG’s mean rate of prescribing of a) gabapentin, b) pregabalin, and c) oxycodone. Missing CCGs are given in white and are excluded due to boundary changes or mergers that occurred during the time period of the study (*n* = 187). CCG = clinical commissioning group. GIS = geographic information system. © Crown copyright and database rights 2019. Ordnance Survey (0) 100019252

Geographical variation in CCGs with the highest average rate of prescribing for gabapentin appears primarily to be within northern and eastern areas of the country (Figure 2). Rates of pregabalin prescribing that exceed 1.5 times the national mean also occur in the northern and eastern regions of England. This pattern is largely mirrored in oxycodone, with the addition of high rates of prescribing along the south-east coastline.

In 2017/2018, 13 of the 15 CCGs with the highest rates of prescriptions per 1000 population of gabapentin, pregabalin, and oxycodone were in the North of England (Table 4). Sunderland CCG is in the top five for each of the drugs in the study.

**Table 4.** CCGs with the highest total number of gabapentin, pregabalin, and oxycodone prescriptions per 1000 population in 2017/2018.

**Gabapentin**	**Pregabalin**	**Oxycodone**
**CCG**	**Number of prescriptions (per 1000 population**)	**CCG**	**Number of prescriptions (per 1000 population**)	**CCG**	**Number of prescriptions (per 1000 population**)
NHS Hardwick	370.2	NHS Durham Dales, Easington and Sedgefield	283.9	NHS Hull	129.0
NHS Blackpool	370.1	NHS Darlington	283.6	NHS Salford	91.0
NHS Durham Dales, Easington and Sedgefield	369.8	NHS Lincolnshire East	283.6	NHS Blackburn with Darwen	87.8
NHS Sunderland	350.7	NHS South Tees	274.8	NHS Sunderland	83.6
NHS Salford	345.9	NHS Sunderland	272.8	NHS Thanet	81.7

CCGs that did not exist in 2013/2014 and 2017/2018 are excluded from this data set. CCG = clinical commissioning group.

## Discussion

### Summary

This retrospective secondary analysis of open source data demonstrates a sustained and significant increase in the rate of prescribing between April 2013 and March 2018 of gabapentin, pregabalin, and oxycodone. A variation in rate of prescribing per capita per GP practice was strongly associated with the deprivation quintile of GP practice, with the practices within the most deprived quintile prescribing more on average of each of the drugs than those in the least deprived quintile. There was a geospatial pattern in CCGs with a rate of prescriptions 1.5 greater than the mean national rate, with predominantly northern and eastern CCGs in this category.

These findings of growing gabapentin and pregabalin prescribing may be a result of greater scrutiny of the prescribing of tramadol and other opioid-based analgesics, as a result of the reclassification of tramadol in 2014 as a Schedule 3 controlled drug.^22^ Historically, the side-effect and addiction profiles of the gabapentin and pregabalin medications are less well known. While the evidence around gabapentin and pregabalin misuse is now established, the extent to which such misuse may be less harmful in comparison to opioid misuse needs now to be understood. Yet oxycodone prescribing continues to increase, albeit to a lesser degree than the gabapentin and pregabalin drugs in this study. The geospatial pattern of CCGs with the highest mean rates of prescribing reflects findings of other literature in the area.

### Strengths and limitations

The dataset is a compilation of three open source datasets, and thus the proportion of GP practices lost through exclusion is likely higher than if there was one definitive dataset. Convenience sampling of excluded GP practices was conducted to ensure suitability. The aggregate nature of the dataset means that key patient-level factors that influence prescribing of DFM may confound the findings. These include individual patient characteristics, cancer and palliative prescribing, GP characteristics, and local prescribing practices. The data included also covers prescriptions dispensed only. The dataset’s exclusion criteria mean the average number of included GP practices within the dataset remained in excess of 88%. The findings of this study are therefore representative of a national picture.

### Comparison with existing literature

These findings broadly align with other published literature in respect to the increase in oxycodone prescriptions, and the prevailing geospatial pattern of high prescribing areas. However, this is the first study to describe and characterise prescription rates and volumes of gabapentin and pregabalin medications alongside an opioid, oxycodone.

The growing perception and reports of increased prescriptions, and therefore the potential for harm from these drugs, are supported by the results of this study. The substantial and significant increases observed in annual gabapentin, pregabalin, and oxycodone prescriptions per capita will contribute to increases in morbidity and mortality related to use and misuse of these drugs. Oxycodone has seen significant increases in prescribing per capita. Given the known potential for harm with this drug, the increases observed support further investigation to understand the underlying causes. In April 2019, the UK Home Office reclassified pregabalin and gabapentin as Schedule 3 drugs. Further analysis following this policy change will ascertain if this legislation will impact rates of prescribing in primary care in future.

The current analysis suggests that the increased prescribing rate of these drugs is not driven by an ageing population alone. There is substantial variation in annual prescribing of gabapentin and pregabalin medications and oxycodone per capita between GP practices based on deprivation quintile; a finding that may reflect the higher prevalence of chronic pain in the most deprived populations.^23^ However, the risk of iatrogenic harm arising from DFM-use among more deprived populations may exacerbate the wider health inequalities already experienced by people living in these areas. Despite alcohol intake being higher in middle and upper socioeconomic populations than the most deprived, the burden of alcohol-related harm is suffered disproportionately by the latter, indicating poorer resilience to substance misuse related harms.^24^ Therefore, vulnerable communities, who are more likely to be prescribed DFM per capita, may be at higher risk of iatrogenic harm from these drugs than people living in more affluent areas.

### Implications for research and practice

GPs in England are limited in the treatment options available for patients with chronic pain. Pressures on specialist pain management and mental health teams means that access to programmes is often restricted. An essential component of harm reduction strategies would be to improve access to specialist services and increase non-pharmacological interventions, such as I-WOTCH.^25^ Further education for both primary care professionals and the public should also be considered.

In conclusion, there have been significant increases in annual prescriptions of gabapentin, pregabalin, and oxycodone per capita in English primary care. The scale of the increases in prescribing of these medications may be driving increased harm in the form of drug-related mortality and morbidity. The experience of the prescription drug misuse epidemic in the US should serve as an early warning to England’s NHS and prompt action to better understand the underlying explanations for the increases in prescribing, and the variation in prescribing rates between GPs and between CCGs.
